# Reliability of MRI diagnosis of RAMP lesions with and without a structured checklist: A study of 200 MRIs

**DOI:** 10.1002/jeo2.70789

**Published:** 2026-05-25

**Authors:** Marco Puce, Carlo Minoli, Leonardo Clausetti, Giuseppe Fedele, Paolo Ferrua, Simone Radaelli, Eugenio Uderzo, Pietro Simone Randelli

**Affiliations:** ^1^ UOC Ortopedia e Traumatologia Pediatrica ASST Centro Specialistico Ortopedico Traumatologico Gaetano Pini‐CTO Milan Italy; ^2^ Scuola di Specializzazione in Ortopedia e Traumatologia Università degli Studi di Milano Milan Italy; ^3^ U.O.C. Week Surgery ASST Centro Specialistico Ortopedico Traumatologico Gaetano Pini‐CTO Milan Italy; ^4^ U.O.C. 1 Clinica ortopedica ASST Centro Specialistico Ortopedico Traumatologico Gaetano Pini‐CTO Milan Italy; ^5^ Laboratory of Applied Biomechanics, Department of Biomedical Sciences for Health Università Degli Studi di Milano Milan Italy; ^6^ Department of Biomedical Sciences for Health Research Center for Adult and Pediatric Rheumatic Diseases (RECAP‐RD) Università degli Studi di Milano Milan Italy

**Keywords:** ACL, knee, meniscus, RAMP, sport medicine

## Abstract

**Purpose:**

This study intended to evaluate the reliability of diagnosing meniscal RAMP lesions on magnetic resonance imaging (MRI) using a newly developed identification checklist. The primary objective was to standardise MRI interpretation to reduce operator‐dependent variability and enhance diagnostic agreement.

**Methods:**

In total, 1350 knee MRI scans were initially reviewed, with 200 matching the predefined inclusion and exclusion criteria. Four sports medicine surgeons were divided into two groups: one using the developed RAMP lesion identification checklist and the other interpreting the MRI scans without it. Interobserver reliability was assessed using Cohen's kappa coefficient at two distinct time points. After 1 month, both groups reassessed the MRI scans using the checklist. The Intraclass Correlation Coefficient (ICC) was also calculated to determine the overall diagnostic consistency between the groups.

**Results:**

The introduction of the checklist significantly improved interobserver reliability. In the initial evaluation, the group using the checklist exhibited a higher level of diagnostic agreement (Cohen's kappa = 0.779, 89% agreement) compared to the group without the checklist (Cohen's kappa = 0.057, 60.5% agreement). After 1 month, when both groups used the checklist, diagnostic concordance improved substantially (Cohen's kappa = 0.698, 85% agreement). The ICC between the groups indicated strong diagnostic consistency (0.759), further highlighting the checklist's effectiveness.

**Conclusions:**

The RAMP lesion identification checklist significantly enhances the reliability of MRI‐based diagnoses by standardising the interpretation process and reducing observer variability. This protocol offers significant value in the preoperative setting by enhancing diagnostic consensus and surgeon confidence, ultimately allowing for more comprehensive surgical planning. However, further validation studies comparing MRI findings with direct arthroscopic evaluations are necessary to confirm its clinical utility.

**Level of Evidence:**

Level 3.

**Abbreviations**
ACL: anterior cruciate ligament
ICC: Intraclass Correlation Coefficient
MRI: magnetic resonance imaging
PD: proton density
STIR: Short‐Tau Inversion Recovery

## INTRODUCTION

RAMP lesions refer to disruptions of the meniscocapsular or meniscotibial attachments of the posterior horn of the medial meniscus, typically located in the posteromedial compartment [8].

The posterior attachment of the medial meniscus is particularly vulnerable due to the presence of a fat pad situated between the stronger meniscal and capsular structures and the semimembranosus tendon, which inserts posteriorly to the posterior horn of the meniscus [4, 9]. During the traumatic event, reflexive contraction of the semimembranosus caused by anterior tibial subluxation results in detachment between the meniscus, which translates anteriorly, and the capsule, which is pulled posteriorly by the semimembranosus tendon [14].

RAMP lesions increase anterior tibial translation, dynamic rotational laxity and excessive rotational movement of the knee. If left unaddressed during anterior cruciate ligament (ACL) reconstruction, these factors can exacerbate biomechanical instability and contribute to persistent tibiofemoral laxity [18, 19].

The reported incidence of RAMP lesions in patients with ACL tears ranges between 9% and 55% [2], highlighting the diagnostic challenges due to variability in identification. Although arthroscopy remains the diagnostic gold standard, specific lesions, particularly hidden types (Type 3, according to the Thaunat classification) [22], may not be easily visualised. In these cases, meniscal mobility during probing may raise suspicion [5, 9, 10, 16]. However, these additional diagnostic manoeuvres are seldom routinely performed during standard ACL reconstructions unless there is a preoperative suspicion of a RAMP lesion, emphasising the need for accurate radiological assessment.

Magnetic resonance imaging (MRI) is the preferred radiological exam for identifying RAMP lesions [21], with a reported accuracy of approximately 95%, including 87.5% for meniscal‐tibial ligament lesions and 96.4% for peripheral meniscal tears [2, 25]. The most informative MRI sequences for detecting RAMP lesions are proton density (PD) fat‐saturation and T2‐weighted images, both with and without fat suppression, in the axial and sagittal planes [15]. However, diagnostic sensitivity remains variable depending on: the field strength, the knee position and the use of dedicated sequences. Additionally, the near‐full extension of the knee during imaging reduces meniscal‐capsular separation, further complicating their identification [13].

In response to these diagnostic challenges, various MRI findings have been suggested as indirect indicators of RAMP lesions [14, 25]:

- Thin fluid signal between the posterior horn of the medial meniscus and the posteromedial capsule.
- Oedema between the proximal posteromedial edge of the tibia and the semimembranosus tendon.
- Posteromedial tibial bone marrow oedema.

This study aims to evaluate whether a structured MRI checklist improves interobserver agreement for the diagnosis of RAMP lesions among orthopaedic surgeons and to develop a tool to improve the diagnosis of RAMP lesions on MRI. The hypothesis is that an identification checklist based on primary and secondary MRI signs of RAMP lesions will help standardise image interpretation and reduce operator‐dependent variability.

## MATERIALS AND METHODS

This study received approval from the institutional review board (authorisation: Fondazione IRCCS Ca' Granda Ospedale Maggiore Policlinico ‐ Milan Area 2, Lombardy, Italy ‐ n°848_2021, dated 14 September 2021).

All knee MRIs performed at the research Institution between January 2018 and July 2023 were screened, including only those acquired using a high‐field strength (1.5 T) scanner. The analysis focused on T2‐weighted Short‐Tau Inversion Recovery (STIR) and PD‐weighted sequences in the coronal, sagittal and axial planes.

As for the inclusion criteria, patients aged between 18 and 35 years were selected with a confirmed ACL injury, verified through both radiological reports and MRI evaluation.

Exclusion criteria: all patients under 18 or over 35 years of age, patients with neoplastic conditions affecting the knee, patients with multiligamentous knee injuries, patients with radiological evidence of previous surgeries or injuries to the affected limb and patients with open physes.

Patients outside this age range were excluded to reduce heterogeneity in meniscal morphology and because very young or older patients rarely undergo arthroscopic evaluation for RAMP pathology, limiting the applicability of validation.

Based on these criteria, 200 knee MRIs were included in the study.

**Figure 1:**
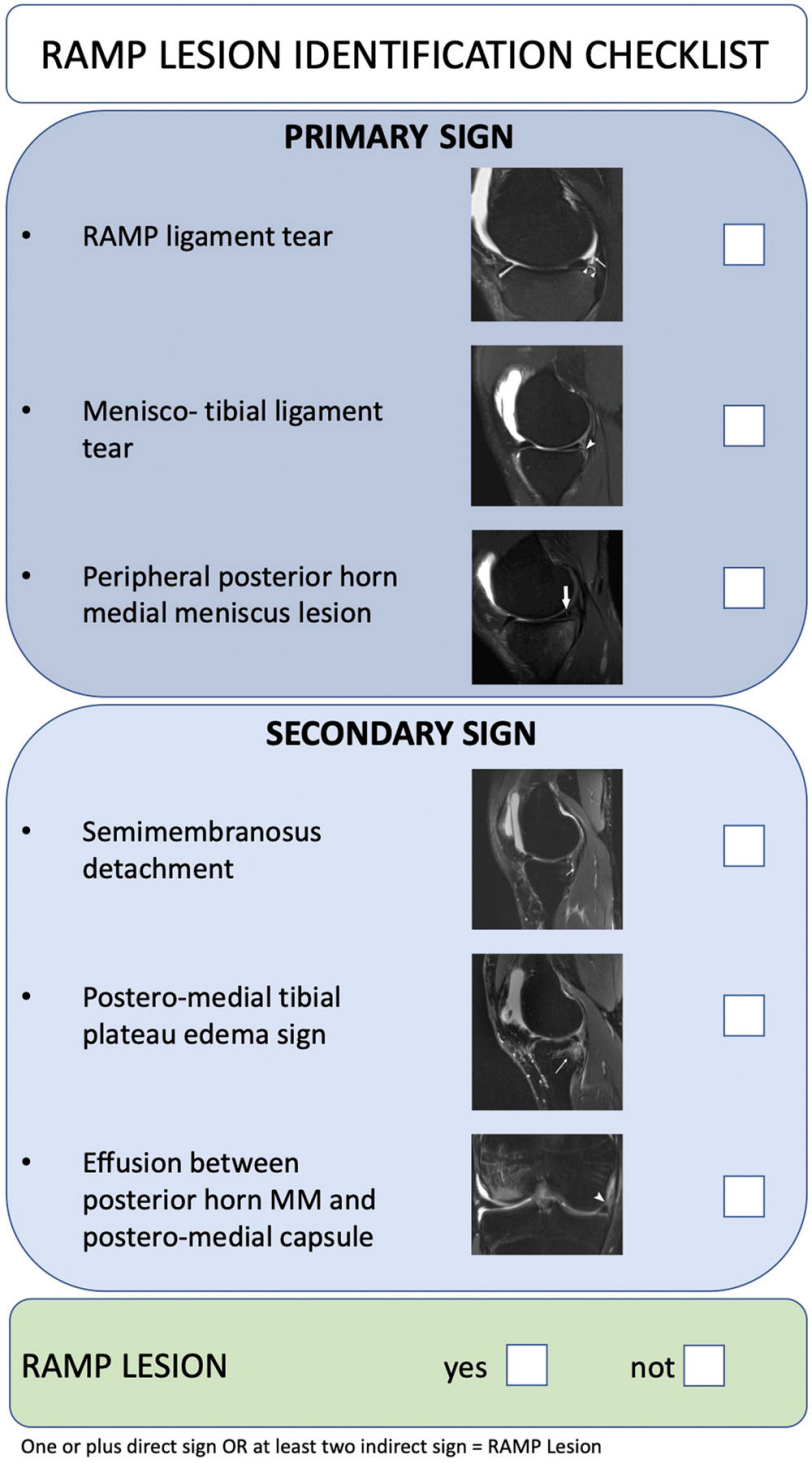
RAMP lesion identification checklist.

A RAMP lesion identification checklist (Figure 1) was developed to standardise the diagnostic process. It was designed as an expert‐derived, preliminary standardisation instrument intended for orthopaedic surgeons who routinely perform preoperative MRI assessments, drawing on criteria from the existing literature regarding both direct and indirect MRI signs of RAMP lesions [25]. The checklist included items corresponding to key parameters for the diagnosis of RAMP lesions: meniscus‐capsular tear, meniscotibial ligament tear and peripheral tear of the posterior horn of the medial meniscus. Additionally, indirect signs such as fluid interposition between the proximal posteromedial edge of the tibia and semimembranosus tendon, oedema in the posteromedial tibial plateau and the presence of effusion or fluid between the posterior horn of the medial meniscus and the posteromedial joint capsule were included: one or plus direct signs OR at least two indirect signs, meaning a RAMP lesion can be diagnosed.

Four orthopaedic surgeons with a special focus on sports medicine participated in the study, reviewing the 200 knee MRIs. The surgeons were randomly divided into two groups. At the initial time point (T0), the first group of two surgeons reviewed the MRIs using the checklist, while the second group conducted their assessments without the checklist. Interobserver reliability was calculated using Cohen's kappa coefficient and percentage agreement. Two of the senior investigators are regular attendings in a Sports Traumatology Unit with 5 years of practice, and the other two were residents respectively at their second and fourth year of residency in the same unit.

The checklist was designed as a practical clinical tool intended primarily for orthopaedic surgeons who routinely read MRIs preoperatively.

After 1 month, both groups blindly reassessed the MRIs, this time both utilising the checklist. Interobserver reliability was recalculated, and the Intraclass Correlation Coefficient (ICC) was determined to assess diagnostic consistency between the groups.

### Statistical analysis

The statistical analysis of this study was performed to evaluate the reliability of RAMP lesion diagnosis using MRI with and without a RAMP lesion identification checklist. The analysis focused on interobserver reliability, diagnostic accuracy and overall agreement between the two groups of observers.

The data collected were primarily categorical (presence or absence of RAMP lesions) and continuous variables (age). Categorical variables such as the presence of RAMP lesions were expressed as percentages, while continuous variables such as age were expressed as the mean and interquartile range. Interobserver agreement was assessed using both Cohen's kappa statistic and the ICC, both standard methods for evaluating reliability in diagnostic studies.

The sample size of 200 MRIs was determined based on the estimated prevalence of RAMP lesions in previous literature, which is reported to range from 9% to 55%. To detect a significant difference between the two groups with 80% power and a confidence level of 95%, we calculated a required sample size based on a presumed prevalence of 40%, with an expected sensitivity and specificity of 0.80 for both measures. This led to the inclusion of 200 MRIs that met the eligibility criteria after the application of the inclusion and exclusion filters.

## RESULTS

**Figure 2:**
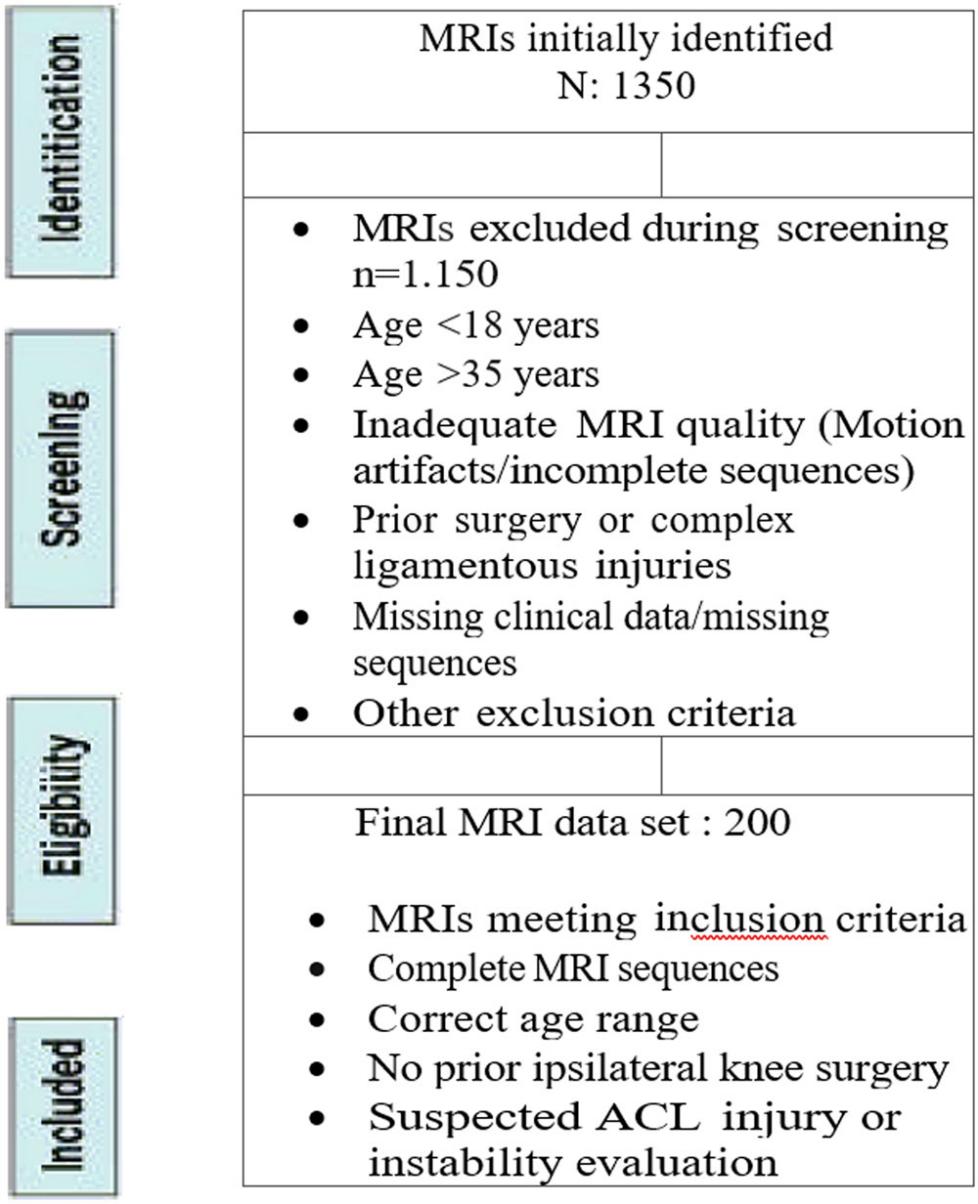
Selection flowchart. ACL, anterior cruciate ligament; MRI, magnetic resonance imaging.

A total of 1350 knee MRIs were reviewed, with 200 meeting the inclusion and exclusion criteria (Figure 2). The study population consisted of 139 males (69.5%) and 61 females (30.5%), with a mean age of 25.75 years. The affected knee was the right one in 109 cases (54.5%), and it was the left one in 91 cases (45.5%).

At T0, the two surgeons in the first group (using the checklist) identified 105 (52.5%) and 108 (54%) RAMP lesions from the 200 MRIs, respectively. In contrast, the second group (without the checklist) identified 44 (22%) and 77 (38.5%) RAMP lesions, respectively.

Interobserver reliability for the first group (using the checklist) at time zero demonstrated strong agreement, with a Cohen's kappa of 0.779 and 89% diagnostic concordance. Conversely, the second group (without the checklist) showed very low agreement, with a Cohen's kappa of 0.057 and 60.5% diagnostic concordance.

After 1 month, when the second group reassessed, the MRIs using the checklist, their identification rates increased to 109 (54.5%) and 107 (52.5%) RAMP lesions, respectively. This change significantly improved their interobserver reliability, with a Cohen's kappa of 0.698 and an agreement of 85%.

At T0, the two surgeons in the first group (using the checklist) identified 105 (52.5%) and 108 (54%) RAMP lesions from the 200 MRIs, respectively. In contrast, the second group (without the checklist) identified 44 (22%) and 77 (38.5%) RAMP lesions, respectively.

Interobserver reliability for the first group (using the checklist) at time zero demonstrated strong agreement, with a Cohen's kappa of 0.779 and 89% diagnostic concordance. Conversely, the second group (without the checklist) showed very low agreement, with a Cohen's kappa of 0.057 and 60.5% diagnostic concordance.

After 1 month, when the second group reassessed, the MRIs using the checklist, their identification rates increased to 109 (54.5%) and 107 (52.5%) RAMP lesions, respectively. This change significantly improved their interobserver reliability, with a Cohen's kappa of 0.698 and an agreement of 85%.

Finally, the overall ICC between the two groups was 0.759, indicating good agreement among the observers, with a percentage agreement of approximately 80%.

## DISCUSSION

The study sought to determine whether the proposed checklist was a reliable tool to detect medial meniscus RAMP lesions and useful in planning every ACL surgery. It also intended to address the operator‐dependent variability in interpreting MRI images for diagnosing RAMP lesions.

The use of this standardised checklist allowed for greater consistency in diagnosing RAMP lesions on MRI. The results demonstrate that, at T0, the surgeons in the first group who used the checklist achieved a high degree of agreement in diagnosing RAMP lesions, with a Cohen's kappa of 0.779 and 89% concordance.

This level of interobserver reliability underscores the efficacy of the checklist in standardising MRI interpretation. In contrast, the second group, which did not use the checklist, showed significantly lower agreement (Cohen's kappa of 0.057 and 60.5% concordance), despite having similar expertise.

After 1 month, the second group reassessed the MRIs using the checklist, resulting in a marked improvement in agreement (Cohen's kappa of 0.698 and 85% concordance). When comparing the overall diagnostic reliability between the two groups, the ICC was 0.759, with an approximate 80% agreement, further emphasising the checklist's role in enhancing diagnostic consistency.

The prevalence of RAMP lesions reported in the literature ranges from 9% to 55% in association with ACL injuries, underscoring the challenges in achieving accurate diagnosis of these lesions [5, 6, 16]. This variability stems from the inherent challenges in detecting RAMP lesions, exacerbated by operator‐dependent limitations [5, 6]. Arthroscopy remains the gold standard for diagnosis, mainly when using specialised techniques like creating a posteromedial portal or employing a 70° arthroscope to better visualise the posteromedial compartment [3, 7, 12]. However, these techniques are not routinely performed during ACL reconstruction, which may contribute to the underdiagnosis of RAMP lesions in clinical practice and the high variability in the incidence of lesions reported in literature [13, 17].

Sonnery‐Cottet et al. reported the highest incidence of RAMP lesions (40%) by systematically creating a posteromedial portal and probing the posteromedial meniscus during ACL reconstruction [20]. Other studies, which did not utilise this approach, reported significantly lower rates of RAMP lesions, suggesting that the lack of routine exploration may contribute to lower detection rates [7, 15, 23, 24]. In this study, the incidence of RAMP lesions was considerably high, probably due to the fact that not all the lesions that are seen in an MRI are consistent with the arthroscopic findings. This may suggest that the checklist raises the threshold for suspecting a lesion, consequently leading to arthroscopic exploration to confirm the suspicion and treatment of the lesion.

**Figure 3:**
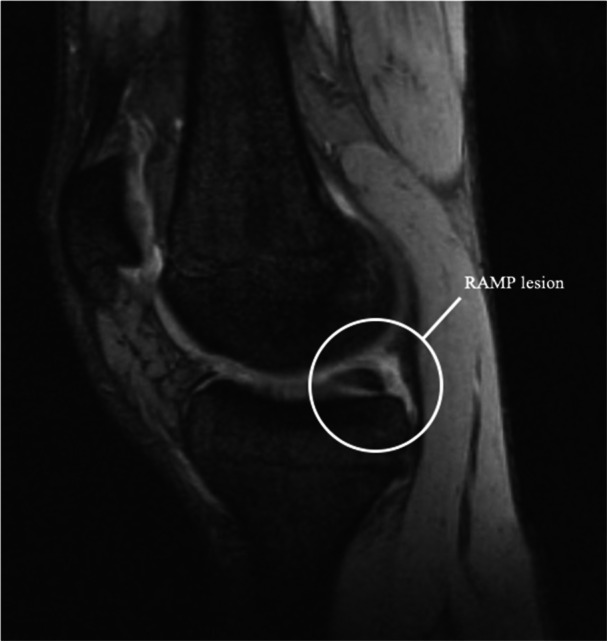
Magnetic resonance imaging (MRI) view: positive for RAMP lesion.

**Figure 4:**
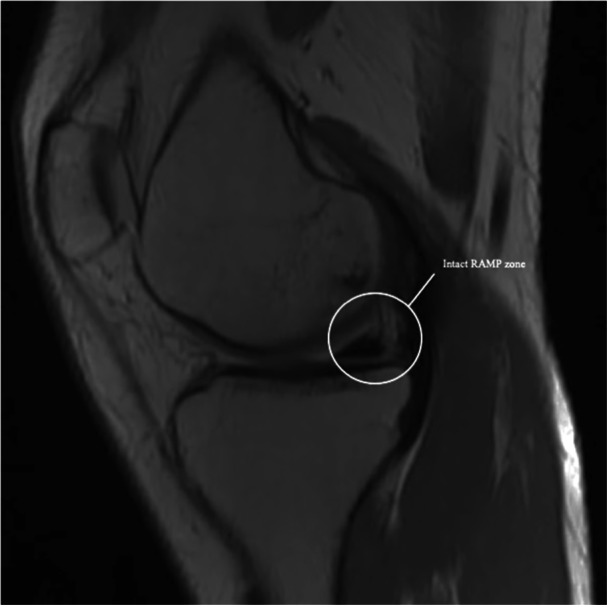
Magnetic resonance imaging (MRI) view: negative for RAMP lesion.

MRI has traditionally been criticised for its limited sensitivity in detecting RAMP lesions, especially when the knee is in full extension, which reduces meniscal‐capsular separation [1, 7, 11, 13, 17, 24] (Figure 3). However, recent studies, such as those by Zappia et al., have demonstrated that MRI accuracy for RAMP lesions (Figures 3 and 4) can reach as high as 95%, with 87.5% accuracy for meniscal‐tibial ligament lesions and 96.4% for peripheral meniscal lesions [25]. The improved diagnostic accuracy of MRI is due not only to advancements in imaging technology but also to the identification of characteristic signs of RAMP lesions, which can be categorised into primary and secondary indicators.

**Figure 5:**
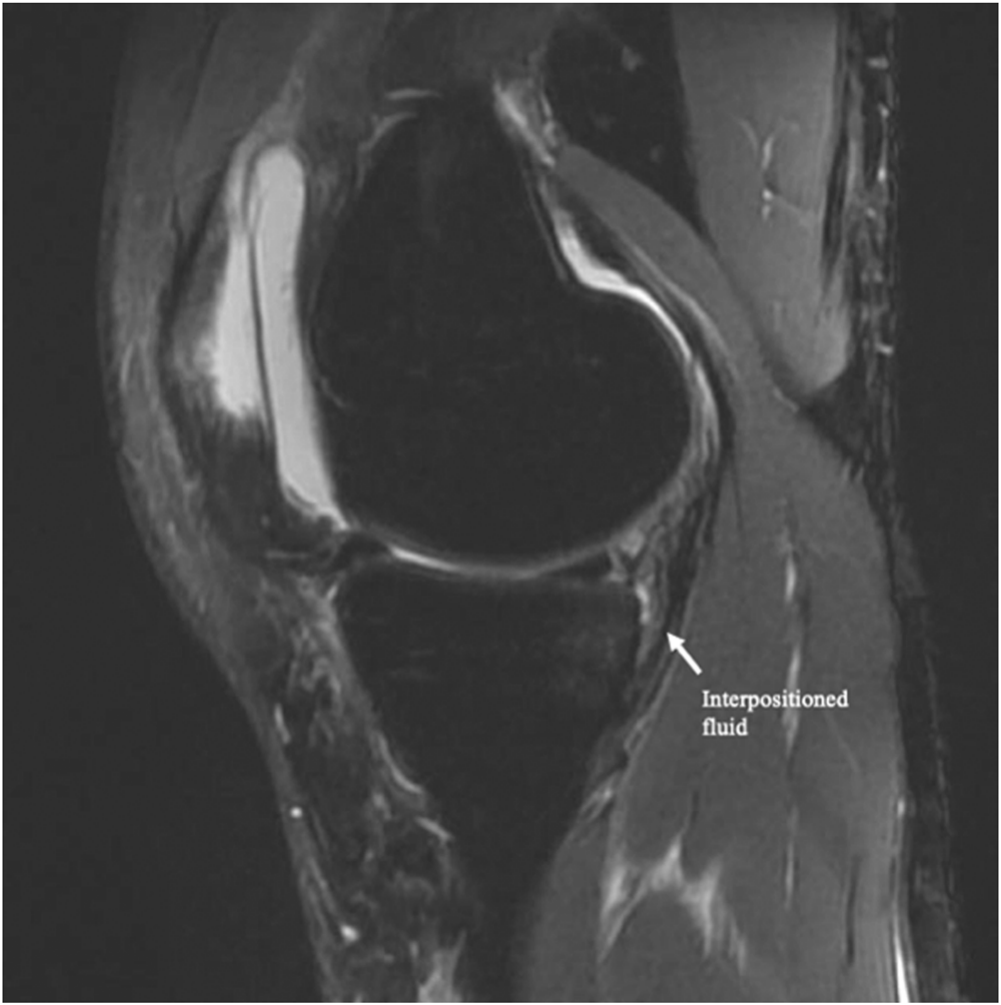
Magnetic resonance imaging (MRI) view: fluid interposition between the tibial edge and semimembranosus tendon.

**Figure 6:**
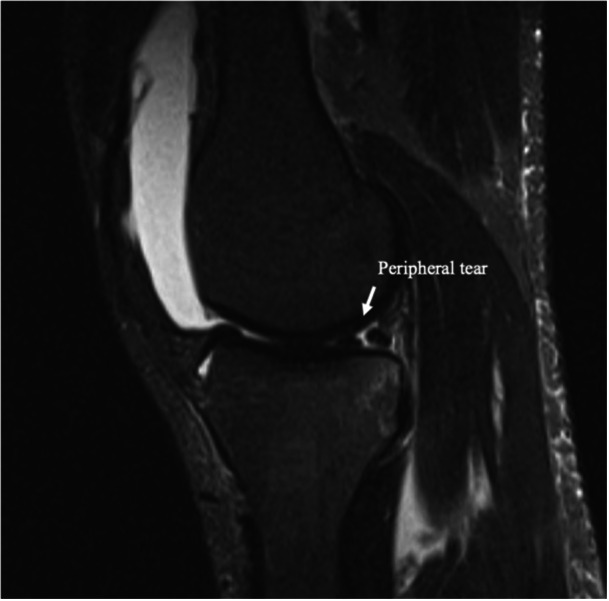
Peripheral meniscal tear in RAMP zone.

**Figure 7:**
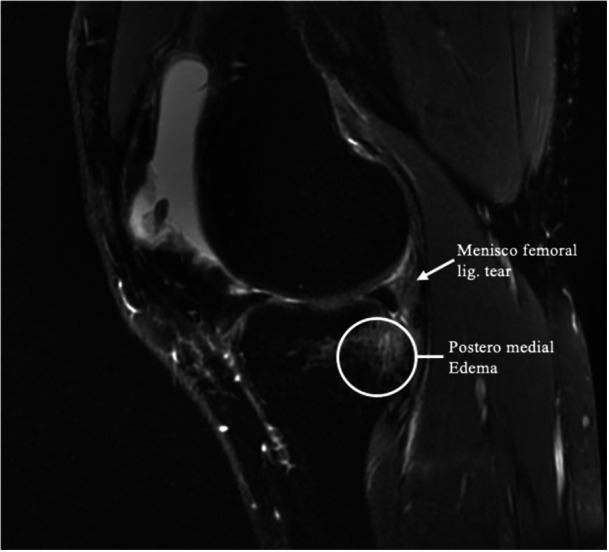
Magnetic resonance imaging (MRI) view: posteromedial oedema and meniscofemoral ligament tear.

Primary signs include high‐intensity fluid signals, on T2‐weighted sequences, that break the meniscal‐femoral ligament, longitudinal mediolateral vertical or oblique tears in the red‐red zone of the posterior horn of the medial meniscus (Figures 5 and 6) and irregularities along the posterior margin of the medial meniscus. Secondary signs include osteochondral fractures, posterior tibial bone marrow oedema (Figure 7) and fluid signal interposed between the posteromedial tibial edge and the semimembranosus tendon. These signs are crucial for improving the detection of RAMP lesions and explaining their increased prevalence in recent literature [7].

The importance of diagnosing RAMP lesions in conjunction with ACL injuries lies in their contribution to anteroposterior and rotational knee instability. Failing to address these lesions during ACL reconstruction can lead to residual instability and increased failure rates. Therefore, standardising the preoperative identification of RAMP lesions using MRI is essential to ensure appropriate surgical planning and intervention [2, 3, 21].

### Limitations

However, several important limitations must be acknowledged. First and foremost, the present study relied exclusively on MRI assessments without systematic comparison to intraoperative arthroscopic findings. While the present findings support the role of a standardised checklist in reducing variability among MRI readers, the lack of arthroscopic validation limits definitive conclusions regarding diagnostic accuracy. As noted by previous authors, agreement between readers can be high even in the absence of a true reference standard, and a reliable tool does not necessarily ensure a high rate of true‐positive findings.

The retrospective design of this study and the lack of standardised, retrievable arthroscopic records for most patients precluded a direct correlation between MRI‐detected RAMP lesions and arthroscopic diagnosis. Therefore, it was not feasible to calculate sensitivity, specificity and predictive values for the checklist in this cohort.

This limitation constrains the strength of clinical conclusions that can be drawn from our results. Although MRI findings can guide preoperative planning and may prompt more careful intraoperative evaluation of the posteromedial compartment, the extent to which checklist‐based MRI diagnoses correspond to true intraoperative lesions remains unproven in this study. Prospective studies that include contemporaneous arthroscopy as a reference standard are needed to evaluate the true diagnostic performance of the checklist.

The second limitation is the absence of data to explore when the MRIs were conducted with respect to the knee injury, hence acute‐phase and chronic MRIs are both considered in this study.

Another limitation is the recall bias, while observing the MRIs for a second time, some images are potentially more memorable than others, hence inducing the investigator to reiterate the previous choice in classifying the lesion.

The last limitation is the lack of radiologists participating in this study, but the checklist was designed as a practical tool intended for young orthopaedic surgeons who routinely read MRIs preoperatively.

Future studies should compare MRI diagnoses with intraoperative findings to validate the effectiveness of the checklist in clinical practice.

## CONCLUSION

The results of this study demonstrate that the use of a structured RAMP lesion identification checklist improves interobserver agreement in the MRI assessment of RAMP lesions. The introduction of the checklist led to a more consistent interpretation of imaging findings, particularly among observers who initially evaluated MRI scans without a standardised approach. These findings suggest that a systematic checklist‐based evaluation can enhance the reproducibility of MRI interpretation for RAMP lesions by providing a clear and uniform framework for assessment. While arthroscopy remains the reference standard for diagnosis, the checklist may represent a useful tool to improve consistency in preoperative MRI evaluation.

## AUTHOR CONTRIBUTIONS

Marco Puce and Carlo Minoli conceived the design of the study. Leonardo Clausetti and Giuseppe Fedele developed the study and performed the computations. Simone Radaelli and Paolo Ferrua verified the analytical methods. Pietro Simone Randelli supervised the findings of this work. All authors discussed the results and contributed to the final manuscript.

## CONFLICT OF INTEREST STATEMENT

The authors declare no conflicts of interest.

## ETHICS STATEMENT

Fondazione IRCCS Ca' Granda Ospedale Maggiore Policlinico ‐ Milan Area 2, Lombardy, Italy ‐ n°848_2021, dated September 14, 2021.

## PATIENT CONSENT STATEMENT

YES.

## PERMISSION TO REPRODUCE MATERIAL FROM OTHER SOURCES

N/A.

## FOR CLINICAL TRIALS

N/A.

## Data Availability

N/A.
